# Dematel-ISM-Based Study of the Impact of Safety Factors on Urban Rail Tunnel Construction Projects

**DOI:** 10.1155/2022/2222556

**Published:** 2022-07-07

**Authors:** Liang Ou, Yun Chen, Jing Zhang, Chongsen Ma

**Affiliations:** School of Traffic & Transportation Engineering, Changsha University of Science and Technology, Hunan 410000, China

## Abstract

The factors affecting urban rail tunnel construction projects are very complex and are influenced by many factors such as the social environment, the construction process, and the way construction is managed. These influencing factors interact with each other, leading to the complexity of the development risks of this type of project. However, at present, the research on engineering construction risk is mainly focused on the field of housing construction, and there are few researches on the risk of urban tunnel construction. At the same time, with the continuous development of urbanization, the coverage of urban underground rail transport is increasing, so it is of great theoretical and practical significance to study the construction process of urban underground tunnels. This paper uses the literature collection method and the LDA model to initially identify the impact factors, and on this basis, the final set of evaluation impact factors is determined by means of expert interviews. Based on the set of influencing factors, the Dematel-ISM model was used to obtain a comprehensive analysis of the factors affecting urban rail tunnel construction projects by comparing topological maps and obtaining a Dematel-ISM model diagram with a cause-effect reachable hierarchy. Finally, the results obtained are applied to the actual development to verify the validity of the model. The results of the study show that construction operation, sequence arrangement, and procedure selection are the key influencing factors for safety risks in urban rail tunnel construction projects.

## 1. Introduction

Transport infrastructure refers to transport engineering facilities that provide public services for social production and residents' lives and is a public service system used to ensure the normal conduct of social and economic activities in a country or region, mainly including railway, highway, aviation, water transport, road and bridge, tunnel, port, and other construction contents. With the continuous development of China's economy, the construction of China's transport infrastructure has made significant developments since 1978. Transport facilities have achieved the transformation from constraining economic development to basically adapting to the level of economic development. By the end of 2020, China's total road mileage reached 5,198,100 kilometers, and railway operating mileage reached 146,300 kilometers, with highways and high-speed railways reaching 161,000 kilometers and 39,800 kilometers, respectively. Both have achieved leapfrog development, and China has also made greater development in shipping. Urban railways, as an important part of the transport infrastructure, have been developed rapidly in recent years. However, urban rail transit inevitably encounters many problems in the construction process, especially in the construction process, as the location of the construction is mostly the city center, and the requirements for construction technology are more stringent. Therefore, this paper will study the risk of tunnel construction in the process of urban rail transit construction, which is of great practical significance.

At present, there are few studies on unsafe behavior and unsafe factors in the construction of urban rail tunnels, and the research content is usually biased towards the purely technical level. Fewer studies have been conducted on the relationship between the risk factors from a management perspective. Therefore, this paper adopts a Dematel-ISM linkage approach to gradually construct an explanatory structural model to understand the key factors and structures affecting the safety of the construction process of urban rail tunnels and to provide suggestions for the control of unsafe behavior in urban rail tunnel construction. Based on the clarification of the key factors and structures affecting the safety of urban rail tunnel construction, ideas and strategies are proposed for the impact of risk-cause pairs.

The Dematel method was developed in the 1970s by Prof. Gabus and Fontela of the Geneva Research Centre and is commonly used to address the relationships between factors in the analysis of complex systems [1]. The method uses mathematical theory to analyze the relationship between the criteria and the strength of their effects by observing the degree of interaction between them and to model the structure of the indicators. In recent years, due to its wide applicability and introduction has received much attention and has been applied in a large number of applications and with the continuous optimization by many scholars, the method has made great progress in the fields of systems engineering, management science, project management, and safety management. In the Dematel method, there are three main expert information expressions as follows: point estimation judgment information, fuzzy number estimation judgment information, and grey number estimation information. Dytczak and Ginda pointed out through experimental analysis that there is a big controversy on how to use the Dematel algorithm and further made suggestions on how to carry out optimization [2]. Bai and Sarkis put forward grey-based Dematel and fused successful key factors for a business project in practical [3]. Lee et al. resolved the infeasibility of Dematel [4]. However, the comprehensive influence matrix (TIM) in Dematel can provide more information than the reachable matrix, so the combination of Dematel and ISM methods can achieve the complementary advantages and integration of the two methods; at the same time, there are also many studies combining Dematel method with ANP, AHP, and VIKOR methods [5–10]. These methods replace the evaluation matrix of the above methods with the constructed TIM matrix, but some scholars point out that the mismatch between the use of Dematel and ANP scales can have some impact on the rationality of the combination of methods [11].

The explanatory structure model (ISM) was proposed by Warfield in 1973, and the method is mainly used to analyze the constituent elements and interdependencies of complex systems. The ISM method forms a top-down arranged hierarchical diagram by decomposing the constituent elements of a complex system and transporting them through the system topology.

This paper further explores the risk influencing factors for sustainable transport infrastructure development in China and analyzes the project using the Dematel method and the ISM method, making the role of the relationship between the fuzzy, entangled risk influencing factors in the development process clear and providing a topological model for the continuous development of transport infrastructure development.

The innovation of this paper is mainly reflected in the following two points:

- This study analyzes the impact of safety factors on urban rail tunnel construction projects based on Dematel-ISM and identifies key impact indicators, which are important for controlling the process of urban rail tunnel construction and ensuring construction safety.
- In this study, the LDA model was used to extract risk factors from the safety factors of previous urban rail tunnel construction projects, which is more scientific and reasonable than traditional methods.

## 2. Influencing Factors and Index System Construction

In order to construct an evaluation index system for transport infrastructure development, the first step of the thesis selects the LDA topic model to carry out topic semantic identification of the 500 highly cited papers collected and selects high-frequency words for risk factor extraction at the best topic count, and the second step is to use the Delphi method to screen the main influencing factors from the preliminary collated risk influencing factors for sustainable development of transport infrastructure.

### 2.1. A Determination of Impact Factor Evaluation Indicators for Sustainable Development of Transport Infrastructure Based on LDA Theme Model

#### 2.1.1. Extraction of Risk Theme Words Based on LDA Model

**Figure 1:**
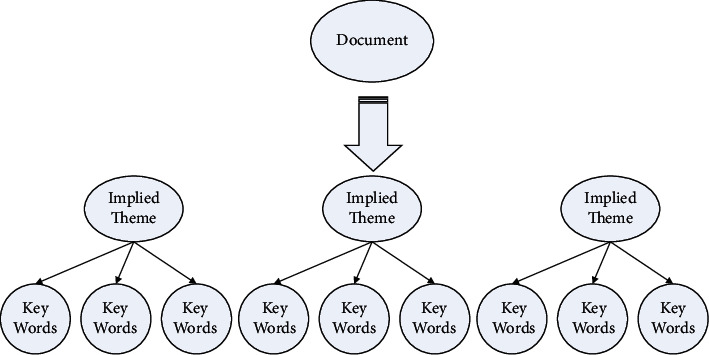
LDA model of the banking theme topology.

The thesis selected the LDA topic model for data mining of selected research texts; data mining is also known as knowledge discovery in databases. By processing a large amount of random data, information containing patterns or values in the text is extracted. Text mining processes textual information and can be used to analyze and refine the themes implied behind the text through technology. Text mining has been used extensively in processing large amounts of irregular text. The thesis chooses latent Dirichlet allocation (LDA) topic model to process selected text data. The LDA model is a commonly used topic model that automatically extracts potential topics from large-scale texts. LDA does not require manual processing of relevant preliminaries during recognition and is an unsupervised machine learning algorithm that can be more efficient in the large-scale text processed. The LDA topic model is artificial in that individual documents are made up of implied topics, and the words in the documents make up the implied topics. The logical structure of the LDA model is shown in Figure 1.

The thesis uses the Gensim and Numpy libraries to construct an LDA topic model after setting up a specialized thesaurus and deactivating words. The LDA topic model is based on a Bayesian model. By counting the input text, the number of words in each of the *M* documents is recorded. The distribution of topics and the distribution of words in each topic are found for each document. The LDA topic model is currently used in information management and user comment analysis.

**Figure 2:**
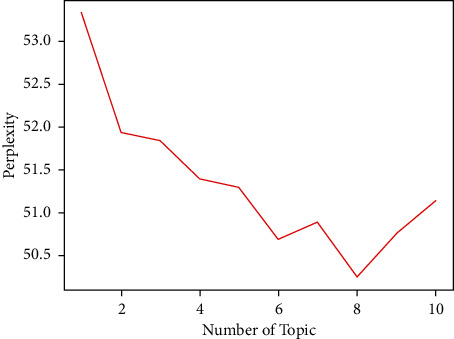
Topic perplexity of LDA model.

As the thesis focuses on Chinese issues, it was searched on CNKI using the keywords “tunnel,” “construction,” and “risk,” and 81 core papers and 16 incident reports with high relevance from 2000 to 2021 were selected as the initial source data. The 81 core papers and 16 incident reports with high relevance from 2000 to 2021 were selected as the initial source data for analysis. After downloading, 55 texts were retained after the initial manual screening. The model perplexity of the analyzed texts is shown in Figure 2.

Through Figure 2, it can be found that the article has the least confusion when the topic is selected as 8. The paper selects topic = 7, 8, 9; analyzes the obtained data; and finds that the best results are obtained when the topic is selected as 8. The results of the analysis of high-frequency topic words in the LDA model part are shown in Table 1.

### 2.2. Preliminary Risk Influencing Factors Determination

Since the thesis addresses the risk of sustainable development of transportation infrastructure, the probability of generation of relevant factors has a small impact on the study. After referring to the subject terms, each subject term is statistically organized and the collated preliminary impact indicators for key risks are shown in Table 2.

The paper identifies the initial risk factors after the subject terms are advanced through the LDA subject matter model. The final evaluation metrics will be determined through the Delphi method. The Delphi method is a commonly used expert scoring method, which was proposed by RAND in 1946. The method collects expert opinions and eventually achieves a unified opinion among the investigators.

Steps of the paper to determine the final evaluation indicators are as follows:

- Clarify the survey objectives, survey purposes, and survey methods and create questionnaires as needed. The method and precautions for filling out the survey questionnaire are explained. The thesis selects the Likert seven-level scale to make the questionnaire. The relative importance among the indicators is judged by means of an expert interview survey. Duplicate indicators and those with less influence were screened out.
- Expert panel members were identified. Ten experts related to theory and practice were selected for this study. Through interviews with experts and questionnaire filling, evaluation indicators are determined based on Table 2. The surveyed experts included government staff, university teachers, relevant researchers, and related practitioners. For the information on survey personnel (see Table 3).
- A questionnaire (see Table 4) was sent to the selected experts by face-to-face communication or letter. And each expert's opinion on the indicators was sought.
- The returned questionnaire results were statistically analyzed, and after collecting the questionnaire data, the questionnaire data were processed using SPSS AU software, and after determining that the data reliability validity met the requirements, the indicators were censored according to the factor loadings. And the final results were returned to each expert.

After the experts discussed the 29 influencing factors, 24 influencing factors with greater practical significance were finally summarized (see Table 5).

## 3. Dematel-ISM Modeling of Safety Influencing Factors in Urban Rail Tunnel Construction Projects

**Figure 3:**
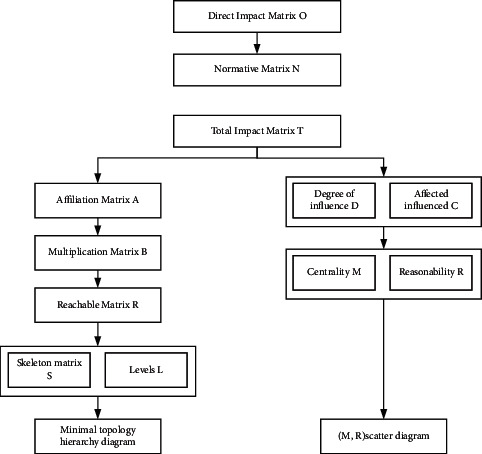
Dematel-ISM modeling of safety influencing factors.

The core idea of this paper is to combine the Dematel method with ISM to reflect the impact of each safety influence factor on the project in the process of urban rail tunnel construction more clearly and reasonably. Compared with text and symbols, the Dematel-ISM method expresses the results more intuitively and clearly and reduces the inverse influence of experts' personal subjective factors on the results on the basis of clear and accurate reflection of the cause-effect relationships seen in each element. In this method, the influencing factors are considered as nodes, and the nodes with causal relationships are linked using directed line segments. In the obtained directed topology diagram, if an element is active, it is called an activity element, and the system containing an Activity element is called extension variable system. In a directed topology diagram, elements are said to form a loop if they are reachable to each other. A reasonable ISM-directed topology diagram requires the presence of a nonloop system. The intercept is set reasonably through the expert interview guided to determine the correlation and hierarchy among the influencing factors and to determine the influence relationship between the factors. The basic process of constructing a model based on the model is as follows (see Figure 3).

### 3.1. Total Impact Matrix Establishment and Specification

Twenty-four influencing indicators of construction safety of urban rail tunnel are quantified by the Dematel method for factor analysis, and on the basis of the quantitative analysis, a direct influence matrix O was formed. The direct impact matrix *O* should be normalized. The formula is as follows:

(1) $$\begin{array}{c} N=\frac{O}{\max}\sum_{j=1}^{n}=O_{ij}={\left(X_{ij}\right)}_{n*n}. \end{array}$$

The normative direct relation matrix *N* is aggregated by accumulating all direct and indirect relationships, and the sum of all direct and indirect relationships of each factor should be identified to obtain the total impact matrix *T* as follows:

(2) $$\begin{array}{c} T=N{\left(I-N\right)}^{-1}, \end{array}$$

where *I* is the unit matrix.

The direct impact *O* matrix of construction safety indicators of the urban rail tunnel is shown in Table 6, and the total impact *T* matrix is shown in Table 7.

### 3.2. The Dematel Factors Analysis

**Figure 4:**
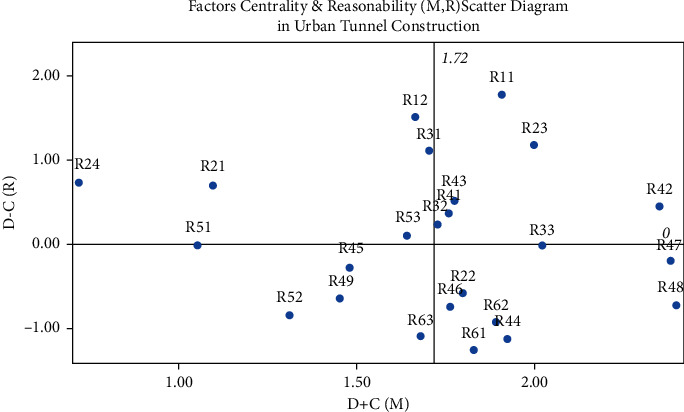
Factors centrality and reasonability (*M*, *R*) scatter diagram.

The cause degree and centrality of each factor of construction safety in the urban rail tunnel should be calculated to obtain the level and weight of each factor. The main steps are as follows:

- Set *D* represents the comprehensive influence of each factor on all other factors. Set *C* represents the comprehensive affected degree of all other factors on each factor.
- Set *D* + *C*(*M*) represents the correlation degree of this factor in the system, which is called centrality. Set *D-C* (*R*) denotes the cause degree of this factor in the system, which is called reasonability. The sets *D*, *C*, *M*, and *R* of construction safety indicators of the urban rail tunnel are shown in Table 8, and the (*M*, *R*) scatter diagram is shown in Figure 4.

*D* + *C* means the significance of the factor centrality in the system; the greater the value, the more important the factor; *D – C* means the influence of a certain factor on other factors. The factor whose value is greater than 0 means more influence on other factors and is denoted as the reason factor. The factor whose value is less than 0 means more influence by others and is denoted as result factor. Each factor should be normalized to obtain weight according to its *D* + *C*(*M*) value.

From the factors analysis of construction safety in the urban rail tunnel, *R*_11_ (stratigraphic conditions), *R*_12_ (complex hydrological situation), *R*_23_ (complex surrounding building conditions), and *R*_31_ (accuracy of survey data) have the highest reasonability, which means the greatest impact on other factors. *R*_48_ (construction operation), *R*_47_ (sequence arrangement), and *R*_42_ (procedure selection) have the highest centrality, which means the fundamental impact on the construction safety of the urban rail tunnel.

### 3.3. ISM Factor Analysis and Reachable Matrix Establishment

Since the Dematel factors analysis on themselves belongs to each factor interaction correspondence, the unit matrix *I* is used to indicate the influence value of factors on themselves. The comprehensive influence relationship of factors in the whole system can be replaced by multiplication matrix *B*.

(3) $$\begin{array}{c} B=T+I. \end{array}$$

In matrix *B*, if *b*_*ij*_ = 0, it shows that factor *b*_*i*_ has no effect on *b*_*j*_; otherwise, *b*_*i*_ has an effect on *b*_*j*._ The matrix *B* obtained in Dematel factors includes not only the existence of factor relationships but also the degree of scope of factors interaction. Therefore, the matrix *B* calculated by Dematel can be simplified to reachable matrix *R*, which shows the reachable hierarchical structure of the urban rail tunnel safety index system.

The reachable matrix *R* is used to reflect a directed topological hierarchy graph that can link each influencing safety factor of an urban rail tunnel through a certain path. The threshold value *λ* = 0.016 is determined according to the actual situation and the influence strength, which the purpose of threshold setting is to discard the less influential relationship and simplify the factor relationship, so as to clearly express the main system structure level. The matrix *R* is simplified according to the following formula. The matrix *R* of the urban rail tunnel safety index system is shown in Table 9.

(4) $$\begin{array}{c} R=\left(r_{ij}\right)=\left\{\begin{array}{c} 0,r_{ij}<\lambda ,\\ 1,r_{ij}\geq \lambda . \end{array}\right. \end{array}$$

### 3.4. Topological Hierarchy Diagram Establishment

There is a reachable set *R*, a prior set *Q*, and a common set *A* from the matrix *R*, where *T* = *R* ∩ *Q* to further get the relationship levels of interaction among factors. All the factors of each row correspond to a path value of 1 are called reachable sets *R*(*i*). All the factors of each corresponding column value of 1 are called the prior set *Q*(*i*). The common set of the reachable set *R*(*i*) ∩ *Q*(*i*) and the prior set is called *T*(*i*). The topological hierarchy extraction process of the urban rail tunnel safety index system can be seen in Table 10.

By comparing directed topological diagrams, the influencing factors of construction safety in the urban rail tunnel are analyzed comprehensively to determine the correlation and hierarchy among the influencing factors and determine the influencing relationship among factors. The basic process of constructing the model according to this is shown in Table 11.

From the topological hierarchy extraction process, the levels can be shown in Table 11.

**Figure 5:**
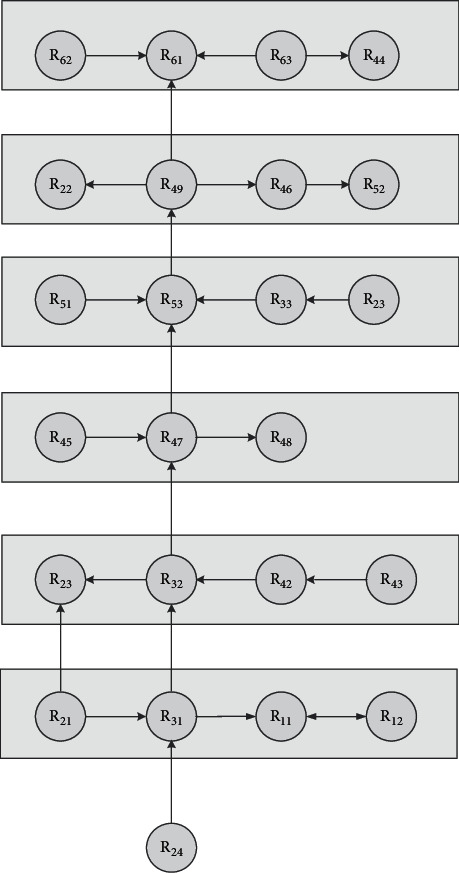
Topological hierarchy diagram of urban rail tunnel safety index.

According to the relationship between the confrontation hierarchy extracted process and levels, the directed topological hierarchy diagram can be drawn as shown in Figure 5.

As shown in Figure 5, the root cause factors set of *R*_24_ (climatic conditions), *R*_11_ (stratigraphic conditions), *R*_12_ (complex hydrological situation), *R*_23_ (complex surrounding building conditions), and *R*_31_ (accuracy of survey data), which are at the bottom of the system and are not affected by other factors, can directly or indirectly affect other factors within the system. The features of factors up to down are from superficial to essential, which means dominant levels are from level 4 to level 7. As shown in figures, factors of *R*_23_ (complex surrounding building conditions), *R*_31_ (accuracy of survey data), *R*_32_ (design reasonableness), *R*_48_ (construction operation), *R*_47_ (sequence arrangement), and *R*_42_ (procedure selection) are the dominant function factors of great importance, which means their fundamental impact on construction safety of urban rail. It can be found that geological conditions, planning and design, and construction technology are more important indicator categories in the safety of urban railway tunnel construction, which should be paid more attention. It also can be noted that by combining the weight analysis of factors in Table 9, *R*_48_ (construction operation), *R*_47_ (sequence arrangement), and *R*_42_ (procedure selection) have the greatest centrality and importance for the construction safety of urban rail, which means that the choice of technology and the effectiveness of its implementation during construction have a dominant influence on construction safety in urban railways.

## 4. Conclusions

This paper studies the risk of tunnel construction in the process of urban rail transit construction. It adopts a Dematel-ISM linkage approach to gradually construct an explanatory structural model to understand the key factors and structures affecting the safety of the construction process of urban rail tunnels. This paper uses the literature collection method and the LDA model to initially identify the impact factors, and on this basis, the final set of evaluation impact factors is determined by means of expert interviews. Based on the research 6 clarification of the 24 key factors affecting the safety of urban rail tunnel construction can be obtained, then the Dematel-ISM model was used to obtain a comprehensive analysis of the factors affecting urban rail tunnel construction projects by comparing topological maps and obtaining a Dematel-ISM model diagram with a cause-effect reachable hierarchy. Finally, the results obtained are applied to the actual development to verify the validity of the model.

From the research, it can be found that the root cause factors set of *R*_24_ (climatic conditions), *R*_11_ (stratigraphic conditions), *R*_12_ (complex hydrological situation), *R*_23_ (complex surrounding building conditions), and *R*_31_ (accuracy of survey data), which are at the bottom of the system and are not affected by other factors, can directly or indirectly affect other factors and result within the system, which should be paid more attention. It can be found especially during the prior period that the most direct safety impact factors come from geological conditions, climatic conditions, and complex surrounding conditions. Therefore, on the basis of fully collecting and possessing accurate information, professional experts should be invited to improve the accuracy and rationality of conditions research in this phrase. During the prior period, the possible changes in the conditions of the project shall be fully considered, and the disposal plan shall be made for relevant changes to reduce the possible impact of unpredictable changes in conditions.

It also notes reachable hierarchy diagram that *R*_31_ (accuracy of survey data), *R*_32_ (design reasonableness), *R*_48_ (construction operation), *R*_47_ (sequence arrangement), and *R*_42_ (procedure selection) are the dominant function factors of great importance, which means their fundamental impact on construction safety of urban rail. It can be found that planning and design and construction technology are more important indicator categories in the safety of urban railway tunnel construction, which should be paid more attention. Design and construction periods are also important for the safety of the urban railway tunnel. Combing the weight analysis of factors, *R*_48_ (construction team arrangement), *R*_47_ (sequence arrangement), and *R*_42_ (procedure selection) have the greatest centrality for construction safety of urban rail, and the calculations imply that the choice of technology and the effectiveness of its implementation have a dominant influence on construction safety in urban railways during construction, which means we should pay full attention to the construction standards, reasonable construction arrangement, and team arrangement. This paper puts forward the important influencing factors that may have a great impact on the safety of urban railway tunnel construction, which can provide some reference for the construction of relevant projects and improve the investment efficiency. At present, further research is needed on the relationship of factors contained in the study and the impact degree on the project caused by the relationship between them. Therefore, it is necessary to conduct in-depth research on the practical cases of urban railway tunnel construction.

In this study, the risk factors were extracted using the LDA model, and based on this, the relationship between the influencing factors was investigated using the Dematel-ISM method. However, the process of determining the relationships between the factors relies heavily on expert scoring, which is highly subjective, and does not take into account the impact of information technology and industrial clustering on tunnel construction risks.

## Figures and Tables

**Table 1:** Analysis results of some high-frequency subject words in LDA model.

Topic 0		Topic 1		Topic 2		Topic 3	
Word	Prob	Word	Prob	Word	Prob	Word	Prob
Tunnel	0.026	Tunnel	0.018	Engineering	0.011	TBM	0.022
Project	0.020	Shield	0.013	Production	0.007	Construction	0.019
Construction	0.015	Construction	0.013	Municipalities	0.007	Tunneling	0.011
Assessment	0.010	Subway	0.009	Management	0.007	Tunneling	0.009
Grade	0.008	Technology	0.005	Accidents	0.006	Pipeline	0.009
…		…		…		…	

Topic 4		Topic 5		Topic 6		Topic 7	
Word	Prob	Word	Prob	Word	Prob	Word	Prob
Construction	0.022	Production	0.019	Tunnel	0.011	Construction	0.023
Accidents	0.008	Accidents	0.011	House	0.010	Tunnel	0.018
Rebar	0.005	Emergency response	0.009	Waterlogging	0.008	Structure	0.014
Work	0.005	Shield	0.008	Shield	0.007	Deformation	0.013
Management	0.005	Rail transit	0.007	Underground	0.007	Control	0.011
…		…		…		…	

**Table 2:** Statistics of factors influencing sustainability in transportation.

No.	Factor name
1	Stratigraphic conditions
2	Complex hydrological situation
3	Complex underground pipelines
4	Site disturbance
5	Complex surrounding building conditions
6	Poor climatic conditions
7	Accuracy of survey data
8	Design reasonableness
9	Reasonableness of scheme selection
10	Project scale
11	Process selection
12	Tunnel type
13	Construction technology risks
14	Informatization
15	Monitoring and measurement
16	Worker's working condition
17	Work sequence arrangement
18	Equipment use
19	Uncoordinated construction relationship
20	Poor safety awareness
21	Improper installation and commissioning of equipment
22	Improper application of new technology
23	Comprehensive quality of construction unit
24	Construction organization design
25	Safety technical measures
26	Safety hazard rectification
27	Safety education
28	Safety inspection
29	Emergency preparedness

**Table 3:** Basic information of the research experts.

Work area	Number	Title	Average years of service
Government worker	2	—	3
Professoriat	3	Pro.Dr.	15
Researcher	3	PhD	6
Relevant employees	2	Manager	5

**Table 4:** Transportation infrastructure impact relevance score.

Factor name	1	2	3	4	5
Poor stratigraphic conditions					
Complex hydrological situation					
Complex underground pipelines					
Site disturbance					
…					
Safety inspection situation					
Emergency plan					

Note: Please fill in the questionnaire according to the actual situation. Scores 1 to 9 indicate that the risk impact is minimal to large.

**Table 5:** Index system of construction safety in an urban rail tunnel.

Indicator categories	Indicator names	Indicator symbols
Geological conditions	Stratigraphic conditions	*R*_11_
	Complex hydrological situation	*R*_12_

Construction environment	Complex underground pipelines	*R*_21_
	Site disturbance	*R*_22_
	Complex surrounding building conditions	*R*_23_
	Poor climatic conditions	*R*_24_

Planning and design	Accuracy of survey data	*R*_31_
	Design reasonableness	*R*_32_
	Reasonableness of scheme selection	*R*_33_

Construction technology	Project scale	*R*_41_
	Process selection	*R*_42_
	Tunnel type	*R*_43_
	Construction technology risk	*R*_44_
	Informatization	*R*_45_
	Monitoring and measurement	*R*_46_
	Worker operation	*R*_47_
	Work sequence arrangement	*R*_48_
	Equipment use	*R*_49_

Management factors	Uncoordinated construction relationship	*R*_51_
	Poor safety awareness	*R*_52_
	Comprehensive quality of construction unit	*R*_53_

Safety factors	Safety technical measures	*R*_61_
	Safety education	*R*_62_
	Safety inspection situation	*R*_63_

**Table 6:** Direct impact *O* matrix of safety indicators of urban rail tunnel.

	*R*_11_	*R*_12_	*R*_21_	*R*_22_	*R*_23_	*R*_24_	*R*_31_	*R*_32_	*R*_33_	*R*_41_	*R*_42_	*R*_43_	*R*_44_	*R*_45_	*R*_46_	*R*_47_	*R*_48_	*R*_49_	*R*_51_	*R*_52_	*R*_53_	*R*_61_	*R*_62_	*R*_63_
*R*_11_	0	2	4	4	3	0	3	3	4	3	4	3	4	0	3	3	3	1	0	2	1	4	4	4
*R*_12_	3	0	1	2	2	0	3	3	4	2	4	1	2	0	3	3	3	1	0	3	1	4	4	4
*R*_21_	0	0	0	0	1	0	3	2	4	1	2	1	1	0	2	0	3	2	0	0	0	3	3	3
*R*_22_	0	0	0	0	4	0	0	0	1	0	0	0	1	0	4	1	1	1	0	2	1	4	1	1
*R*_23_	0	0	5	0	0	0	4	5	5	3	4	3	4	1	3	1	3	3	1	2	0	2	2	3
*R*_24_	1	3	0	0	1	0	1	0	0	1	3	0	2	0	0	1	3	1	1	2	0	1	1	1
*R*_31_	0	0	0	0	0	0	0	5	5	4	4	4	4	0	4	3	3	1	1	2	0	3	3	3
*R*_32_	0	0	0	3	2	0	0	0	4	3	3	3	3	1	3	1	2	1	0	1	0	1	1	1
*R*_33_	0	0	0	3	3	0	0	3	0	2	5	2	3	1	2	2	2	1	0	1	0	1	1	1
*R*_41_	0	0	0	2	0	0	1	2	1	0	2	4	4	0	3	3	4	1	2	2	1	2	2	2
*R*_42_	0	0	0	4	0	0	0	5	5	3	0	3	3	2	2	3	5	2	1	2	1	4	3	3
*R*_43_	0	0	0	1	0	0	0	3	3	3	4	0	3	0	2	2	5	2	1	2	1	3	3	3
*R*_44_	0	0	0	0	0	0	0	0	1	1	0	1	0	0	0	1	1	0	0	2	0	4	3	2
*R*_45_	0	0	0	1	0	0	0	0	3	1	2	0	0	0	3	2	2	1	1	0	4	0	0	0
*R*_46_	0	0	0	0	0	0	0	0	0	1	0	0	0	4	0	1	0	0	0	4	2	3	3	3
*R*_47_	0	0	0	5	0	0	0	0	0	0	0	0	3	5	4	0	5	4	3	2	4	3	3	3
*R*_48_	0	0	0	3	0	0	0	0	1	0	2	0	3	4	1	3	0	3	4	1	1	2	2	2
*R*_49_	0	0	0	1	0	0	0	0	0	0	0	0	1	0	1	2	4	0	1	1	1	1	1	1
*R*_51_	0	0	0	0	0	0	0	0	0	0	0	0	1	5	1	4	4	1	0	0	2	0	0	0
*R*_52_	0	0	0	0	0	0	0	0	0	0	0	0	0	0	0	0	0	0	0	0	1	3	3	3
*R*_53_	0	0	0	2	0	0	0	0	0	0	0	0	3	4	2	5	4	4	1	0	0	3	3	3
*R*_61_	0	0	0	1	0	0	0	0	0	0	0	0	2	0	0	1	0	0	0	3	1	0	2	2
*R*_62_	0	0	0	3	0	0	0	0	0	0	0	0	3	0	1	3	1	4	0	1	1	1	0	1
*R*_63_	0	0	0	3	0	0	0	0	0	0	0	0	3	0	1	0	0	1	0	1	1	1	1	0

**Table 7:** Total impact *T* matrix of construction safety indicators of urban rail tunnel.

	*R*_11_	*R*_12_	*R*_21_	*R*_22_	*R*_23_	*R*_24_	*R*_31_	*R*_32_	*R*_33_	*R*_41_	*R*_42_	*R*_43_	*R*_44_	*R*_45_	*R*_46_	*R*_47_	*R*_48_	*R*_49_	*R*_51_	*R*_52_	*R*_53_	*R*_61_	*R*_62_	*R*_63_
*R*_11_	0.002	0.032	0.070	0.115	0.066	0.000	0.059	0.082	0.107	0.078	0.102	0.077	0.129	0.033	0.100	0.097	0.108	0.060	0.021	0.080	0.045	0.131	0.124	0.123
*R*_12_	0.048	0.002	0.023	0.080	0.047	0.000	0.056	0.076	0.099	0.058	0.096	0.041	0.090	0.030	0.092	0.091	0.098	0.054	0.019	0.089	0.042	0.120	0.115	0.114
*R*_21_	0.000	0.000	0.002	0.027	0.024	0.000	0.051	0.049	0.083	0.032	0.052	0.031	0.050	0.016	0.055	0.026	0.075	0.053	0.011	0.023	0.013	0.076	0.074	0.074
*R*_22_	0.000	0.000	0.005	0.013	0.067	0.000	0.005	0.009	0.026	0.008	0.009	0.006	0.035	0.014	0.077	0.030	0.031	0.029	0.006	0.050	0.026	0.083	0.036	0.036
*R*_23_	0.000	0.000	0.082	0.044	0.013	0.000	0.071	0.110	0.119	0.077	0.100	0.076	0.119	0.045	0.091	0.059	0.102	0.083	0.035	0.070	0.024	0.086	0.082	0.096
*R*_24_	0.018	0.049	0.004	0.022	0.021	0.000	0.021	0.014	0.018	0.027	0.062	0.011	0.057	0.015	0.019	0.038	0.073	0.033	0.026	0.051	0.013	0.043	0.041	0.041
*R*_31_	0.000	0.000	0.001	0.044	0.011	0.000	0.002	0.101	0.107	0.086	0.091	0.085	0.113	0.030	0.100	0.086	0.094	0.048	0.034	0.069	0.025	0.095	0.092	0.090
*R*_32_	0.000	0.000	0.003	0.075	0.042	0.000	0.004	0.019	0.084	0.064	0.069	0.063	0.081	0.035	0.075	0.043	0.065	0.038	0.013	0.042	0.017	0.052	0.047	0.046
*R*_33_	0.000	0.000	0.005	0.077	0.057	0.000	0.005	0.067	0.026	0.049	0.098	0.049	0.083	0.037	0.061	0.059	0.067	0.040	0.014	0.043	0.018	0.054	0.048	0.048
*R*_41_	0.000	0.000	0.001	0.061	0.007	0.000	0.017	0.044	0.034	0.014	0.048	0.074	0.098	0.026	0.074	0.077	0.098	0.042	0.047	0.059	0.036	0.070	0.066	0.064
*R*_42_	0.000	0.000	0.001	0.105	0.015	0.000	0.002	0.094	0.103	0.065	0.026	0.064	0.095	0.061	0.070	0.086	0.122	0.065	0.035	0.066	0.042	0.109	0.088	0.086
*R*_43_	0.000	0.000	0.001	0.052	0.009	0.000	0.002	0.062	0.067	0.062	0.081	0.015	0.087	0.026	0.060	0.065	0.115	0.059	0.032	0.060	0.036	0.086	0.082	0.081
*R*_44_	0.000	0.000	0.000	0.012	0.002	0.000	0.000	0.003	0.019	0.019	0.005	0.019	0.014	0.006	0.008	0.026	0.025	0.010	0.004	0.042	0.007	0.075	0.060	0.043
*R*_45_	0.000	0.000	0.000	0.035	0.005	0.000	0.001	0.008	0.056	0.023	0.041	0.006	0.020	0.021	0.065	0.053	0.055	0.033	0.026	0.014	0.077	0.022	0.019	0.019
*R*_46_	0.000	0.000	0.000	0.014	0.001	0.000	0.000	0.001	0.005	0.018	0.004	0.002	0.014	0.072	0.012	0.029	0.012	0.012	0.005	0.073	0.044	0.062	0.062	0.061
*R*_47_	0.000	0.000	0.001	0.105	0.007	0.000	0.001	0.002	0.012	0.006	0.009	0.003	0.076	0.107	0.090	0.034	0.109	0.089	0.062	0.056	0.088	0.082	0.077	0.076
*R*_48_	0.000	0.000	0.000	0.070	0.006	0.000	0.001	0.005	0.027	0.007	0.039	0.005	0.070	0.085	0.038	0.072	0.029	0.067	0.074	0.034	0.037	0.058	0.053	0.052
*R*_49_	0.000	0.000	0.000	0.028	0.002	0.000	0.000	0.001	0.003	0.002	0.003	0.001	0.028	0.014	0.025	0.044	0.074	0.012	0.024	0.025	0.025	0.030	0.028	0.028
*R*_51_	0.000	0.000	0.000	0.017	0.002	0.000	0.000	0.001	0.008	0.003	0.007	0.001	0.030	0.099	0.032	0.081	0.082	0.032	0.012	0.010	0.048	0.016	0.015	0.014
*R*_52_	0.000	0.000	0.000	0.008	0.001	0.000	0.000	0.000	0.001	0.000	0.000	0.000	0.009	0.002	0.004	0.006	0.004	0.006	0.001	0.005	0.020	0.053	0.053	0.053
*R*_53_	0.000	0.000	0.000	0.058	0.004	0.000	0.000	0.002	0.009	0.004	0.007	0.002	0.072	0.086	0.054	0.103	0.089	0.086	0.030	0.020	0.023	0.074	0.071	0.070
*R*_61_	0.000	0.000	0.000	0.023	0.002	0.000	0.000	0.000	0.002	0.001	0.001	0.001	0.040	0.004	0.006	0.022	0.006	0.007	0.002	0.054	0.021	0.011	0.041	0.040
*R*_62_	0.000	0.000	0.000	0.060	0.004	0.000	0.000	0.001	0.004	0.002	0.002	0.002	0.060	0.011	0.028	0.059	0.031	0.075	0.007	0.028	0.026	0.034	0.015	0.030
*R*_63_	0.000	0.000	0.000	0.053	0.004	0.000	0.000	0.001	0.003	0.002	0.001	0.001	0.054	0.004	0.022	0.007	0.006	0.021	0.002	0.024	0.020	0.028	0.025	0.008

**Table 8:** Dematel factors analysis of construction safety in urban rail tunnel.

	*D*	*C*	*M*	*R*	Weight
*R*_11_	1.839	0.069	1.908	1.771	0.046
*R*_12_	1.581	0.083	1.664	1.498	0.040
*R*_21_	0.897	0.201	1.098	0.696	0.026
*R*_22_	0.601	1.198	1.799	−0.596	0.044
*R*_23_	1.581	0.418	1.999	1.163	0.048
*R*_24_	0.720	0.000	0.720	0.720	0.018
*R*_31_	1.406	0.297	1.703	1.109	0.041
*R*_32_	0.976	0.751	1.727	0.224	0.042
*R*_33_	1.003	1.021	2.024	−0.018	0.049
*R*_41_	1.056	0.706	1.761	0.350	0.042
*R*_42_	1.400	0.953	2.353	0.447	0.057
*R*_43_	1.139	0.636	1.775	0.503	0.043
*R*_44_	0.399	1.526	1.925	−1.128	0.047
*R*_45_	0.600	0.882	1.482	−0.282	0.036
*R*_46_	0.506	1.258	1.764	−0.753	0.043
*R*_47_	1.090	1.294	2.384	−0.204	0.057
*R*_48_	0.829	1.570	2.400	−0.741	0.058
*R*_49_	0.398	1.055	1.453	−0.657	0.035
*R*_51_	0.512	0.542	1.053	−0.030	0.026
*R*_52_	0.226	1.086	1.312	−0.860	0.032
*R*_53_	0.868	0.774	1.643	0.094	0.040
*R*_61_	0.283	1.548	1.831	−1.265	0.044
*R*_62_	0.482	1.413	1.894	−0.931	0.046
*R*_63_	0.287	1.395	1.682	−1.109	0.041

**Table 9:** Reachable matrix *R* of urban rail tunnel safety index system.

	*R*_11_	*R*_12_	*R*_21_	*R*_22_	*R*_23_	*R*_24_	*R*_31_	*R*_32_	*R*_33_	*R*_41_	*R*_42_	*R*_43_	*R*_44_	*R*_45_	*R*_46_	*R*_47_	*R*_48_	*R*_49_	*R*_51_	*R*_52_	*R*_53_	*R*_61_	*R*_62_	*R*_63_
*R*_11_	1	1	0	0	1	0	1	1	1	1	1	1	1	1	1	1	1	1	1	1	1	1	1	1
*R*_12_	1	1	1	1	1	0	1	1	1	1	1	1	1	1	1	1	1	1	0	1	1	1	1	1
*R*_21_	0	0	1	1	1	0	1	1	1	1	1	1	1	0	1	1	1	1	0	1	0	1	1	1
*R*_22_	0	0	0	1	1	0	0	0	1	0	0	0	1	0	1	1	1	1	0	1	1	1	1	1
*R*_23_	0	0	1	1	1	0	1	1	1	1	1	1	1	1	1	1	1	1	1	1	1	1	1	1
*R*_24_	1	1	0	1	1	1	1	0	0	1	1	0	1	0	0	1	1	1	1	1	0	1	1	1
*R*_31_	0	0	0	1	0	0	1	1	1	1	1	1	1	1	1	1	1	1	1	1	1	1	1	1
*R*_32_	0	0	0	1	1	0	0	1	1	1	1	1	1	1	1	1	1	1	0	1	0	1	1	1
*R*_33_	0	0	0	1	1	0	0	1	1	1	1	1	1	1	1	1	1	1	0	1	0	1	1	1
*R*_41_	0	0	0	1	0	0	0	1	1	1	1	1	1	1	1	1	1	1	1	1	1	1	1	1
*R*_42_	0	0	0	1	0	0	0	1	1	1	1	1	1	1	1	1	1	1	1	1	1	1	1	1
*R*_43_	0	0	0	1	0	0	0	1	1	1	1	1	1	1	1	1	1	1	1	1	1	1	1	1
*R*_44_	0	0	0	0	0	0	0	0	0	0	0	0	1	0	0	1	1	0	0	1	0	1	1	1
*R*_45_	0	0	0	1	0	0	0	0	1	1	1	0	1	1	1	1	1	1	1	0	0	1	0	0
*R*_46_	0	0	0	0	0	0	0	0	0	0	0	0	0	1	1	1	0	0	0	1	1	1	1	1
*R*_47_	0	0	0	1	0	0	0	0	0	0	0	0	1	1	1	1	1	1	1	1	1	1	1	1
*R*_48_	0	0	0	1	0	0	0	0	1	0	1	0	1	1	1	1	1	1	1	1	1	1	1	1
*R*_49_	0	0	0	1	0	0	0	0	0	0	0	0	1	0	1	1	1	1	1	1	1	1	1	1
*R*_51_	0	0	0	0	0	0	0	0	0	0	0	0	1	1	1	1	1	1	1	0	1	0	0	0
*R*_52_	0	0	0	0	0	0	0	0	0	0	0	0	0	0	0	0	0	0	0	1	1	1	1	1
*R*_53_	0	0	0	1	0	0	0	0	0	0	0	0	1	1	1	1	1	1	1	1	1	1	1	1
*R*_61_	0	0	0	1	0	0	0	0	0	0	0	0	1	0	0	1	0	0	0	1	1	1	1	1
*R*_62_	0	0	0	1	0	0	0	0	0	0	0	0	1	0	1	1	1	1	0	1	1	1	1	1
*R*_63_	0	0	0	1	0	0	0	0	0	0	0	0	1	0	1	0	0	1	0	1	1	1	1	1

**Table 10:** Topological hierarchy extraction process of urban rail tunnel safety index.

*i*	*R*(*i*)	*Q*(*i*)	*A* = *R* ∩ *Q*
*R*11	1, 2, 5, 7, 8, 9, 10, 11, 12, 13, 14, 15, 16, 17, 18, 19, 20, 21, 22, 23, 24	1, 2, 6	1, 2
*R*12	1, 2, 3, 4, 5, 7, 8, 9, 10, 11, 12, 13, 14, 15, 16, 17, 18, 19, 20, 21, 22, 23, 24	1, 2, 6	1, 2
*R*21	3, 4, 5, 7, 8, 9, 10, 11, 12, 13, 15, 16, 17, 18, 20, 22, 23, 24	3, 4, 6	3, 4
*R*22	4, 5, 9, 13, 15, 16, 17, 18, 20, 21, 22, 23, 24	2, 3, 4, 5, 7, 8, 10, 11, 12, 14, 16, 17, 18, 21, 22, 23, 24	4, 5, 16, 17, 18, 21, 22, 23, 24
*R*23	3, 4, 5, 7, 8, 9, 10, 11, 12, 13, 14, 15, 16, 17, 18, 19, 20, 21, 22, 23, 24	1, 2, 3, 4, 5, 6, 8, 9	3, 4, 5, 8, 9,
*R*24	1, 2, 3, 4, 5, 6, 7, 8, 9, 10, 1116, 17, 18, 19, 20, 22, 23, 24	6	6
*R*31	4, 7, 8, 9, 10, 11, 16, 17, 18, 19, 20, 22, 23, 24	1, 2, 3, 5, 6, 7	7
*R*32	4, 5, 6, 8, 9, 10, 11, 12, 13, 14, 15, 16, 17, 18, 20, 22, 23, 24	1, 2, 3, 5, 7, 8, 9, 10, 11, 12,	5, 8, 9, 10, 11, 12,
*R*33	4, 5, 8, 9, 10, 11, 12, 13, 14, 15, 16, 17, 18, 20, 22, 23, 24	1, 2, 3, 4, 5, 7, 8, 9, 10, 11, 12, 14, 17	4, 5, 8, 9, 10, 11, 12, 14, 17,
*R*41	4, 8, 9, 10, 11, 12, 13, 14, 15, 16, 17, 18, 19, 20, 21, 22, 23, 24	1, 2, 3, 5, 7, 8, 9, 10, 11, 12, 14,	8, 9, 10, 11, 12, 14,
*R*42	4, 8, 9, 10, 11, 12, 13, 14, 15, 16, 17, 18, 19, 20, 21, 22, 23, 24	1, 2, 3, 5, 7, 8, 9, 10, 11, 12, 14, 17	8, 9, 10, 11, 12, 14, 17
*R*43	4, 8, 9, 10, 11, 12, 13, 14, 15, 16, 17, 18, 19, 20, 21, 22, 23, 24	1, 2, 3, 5, 7, 8, 9, 10, 11, 12,	8, 9, 10, 11, 12
*R*44	13, 16, 17, 20, 22, 23, 24	1, 2, 3, 4, 5, 7, 8, 9, 10, 11, 12, 13, 14, 16, 17, 18, 19, 21, 22, 23, 24	13, 16, 17, 22, 23, 24
*R*45	4, 9, 10, 11, 13, 14, 15, 16, 17, 18, 19, 22,	1, 2, 5, 7, 8, 9, 10, 11, 12, 14, 15, 16, 17, 19, 21, 23, 24	9, 10, 11, 14, 15, 16, 17, 19,
*R*46	14, 15, 16, 20, 21, 22, 23, 24	1, 2, 3, 4, 5, 7, 8, 9, 10, 11, 12, 14, 15, 16, 17, 18, 19, 21, 23, 24	14, 15, 16, 21, 23, 24
*R*47	4, 13, 14, 15, 16, 17, 18, 19, 20, 21, 22, 23, 24	1, 2, 3, 4, 5, 7, 8, 9, 10, 11, 12, 13, 14, 15, 16, 17, 18, 19, 21, 23	4, 13, 14, 15, 16, 17, 18, 19, 21, 23
*R*48	4, 9, 11, 13, 14, 15, 16, 17, 18, 19, 20, 21, 22, 23, 24	1, 2, 3, 4, 5, 7, 8, 9, 10, 11, 12, 13, 14, 16, 17, 18, 19, 21, 23	4, 9, 11, 13, 14, 16, 17, 18, 19, 21, 23
*R*49	4, 13, 15, 16, 17, 18, 19, 20, 21, 22, 23, 24	1, 2, 3, 4, 5, 7, 8, 9, 10, 11, 12, 14, 16, 17, 18, 19, 21, 23, 24	4, 16, 17, 18, 19, 21, 23, 24
*R*51	13, 14, 15, 16, 17, 18, 19	1, 5, 6, 7, 10, 11, 12, 14, 16, 17, 18, 19, 21	14, 16, 17, 18, 19
*R*52	20, 21, 22, 23, 24	1, 2, 3, 4, 5, 7, 8, 9, 10, 11, 12, 16, 17, 18, 20, 21, 22, 23, 24	20, 21, 22, 23, 24
*R*53	4, 13, 14, 15, 16, 17, 18, 19, 20, 21, 22, 23, 24	1, 2, 4, 5, 7, 10, 11, 12, 15, 16, 17, 18, 19, 20, 21, 22, 23, 24	4, 15, 16, 17, 18, 19, 20, 21, 22, 23, 24
*R*61	4, 13, 16, 20, 21, 22, 23, 24	1, 2, 3, 4, 5, 6, 7, 10, 11, 12, 15, 16, 17, 18, 20, 21, 22, 23, 24	4, 13, 16, 20, 21, 22, 23, 24
*R*62	4, 13, 15, 16, 17, 18, 20, 21, 22, 23, 24	1, 2, 3, 4, 5, 6, 7, 10, 11, 12, 13, 15, 16, 17, 18, 20, 21, 22, 23, 24	4, 13, 15, 16, 17, 18, 20, 21, 22, 23, 24
*R*63	4, 13, 15, 18, 20, 21, 22, 23, 24	1, 2, 3, 4, 5, 6, 7, 10, 11, 12, 13, 15, 16, 17, 18, 20, 21, 22, 23, 24	4, 13, 15, 18, 20, 21, 22, 23, 24

**Table 11:** Hierarchy extraction levels.

Levels	Factors
Level 1	*R*63, *R*62, *R*61, *R*44
Level 2	*R*22, *R*46, *R*49, *R*52
Level 3	*R*51, *R*45, *R*33, *R*53
Level 4	*R*45, *R*47, *R*48
Level 5	*R*32, *R*42, *R*43, *R*23
Level 6	*R*12, *R*11, *R*31, *R*21
Level 7	*R*24

## Data Availability

The raw data supporting the conclusions of this article will be made available by the authors, without undue reservation.
